# Microbial shifts associated to ENSO-derived thermal anomalies reveal coral acclimation at holobiont level

**DOI:** 10.1038/s41598-023-49049-6

**Published:** 2023-12-12

**Authors:** Sandra Montaño-Salazar, Elena Quintanilla, Juan A. Sánchez

**Affiliations:** 1 Division of Microbial Ecology, Department for Microbiology and Ecosystem Science, University of Vienna, Vienna, Austria; 2 Department of Soil and Water Sciences, University of Florida, 2033 Mowry Rd, Gainesville, FL 32610 USA; 3 Laboratory of Marine Molecular Biology (BIOMMAR), Department of Biological Sciences, Universidad de los Andes, Bogotá, Colombia

**Keywords:** Ecology, Microbiology, Molecular biology, Ocean sciences

## Abstract

The coral microbiome conforms a proxy to study effects of changing environmental conditions. However, scarce information exists regarding microbiome dynamics and host acclimation in response to environmental changes associated to global-scale disturbances. We assessed El Niño Southern Oscillation (ENSO)-derived thermal anomalies shifts in the bacterial microbiome of *Pacifigorgia cairnsi* (Gorgoniidae: Octocorallia) from the remote island of Malpelo in the Tropical Eastern Pacific. Malpelo is a hot spot of biodiversity and lacks direct coastal anthropogenic impacts. We evaluated the community composition and predicted functional profiles of the microbiome during 2015, 2017 and 2018, including different phases of ENSO cycle. The bacterial community diversity and composition between the warming and cooling phase were similar, but differed from the neutral phase. Relative abundances of different microbiome core members such as *Endozoicomonas* and *Mycoplasma* mainly drove these differences. An acclimated coral holobiont is suggested not just to warm but also to cold stress by embracing similar microbiome shifts and functional redundancy that allow maintaining coral’s viability under thermal stress. Responses of the microbiome of unperturbed sea fans such as *P. cairnsi* in Malpelo could be acting as an extended phenotype facilitating the acclimation at the holobiont level.

## Introduction

Climate change negatively impacts coral ecosystems globally, causing dramatic ecological transformations^1^. Increasing seawater temperatures associated with global warming represent a severe threat to corals (either Scleractinia or Octocorallia) worldwide since there are upper thermal limits that once exceeded, corals experience significant declines^2,3^. Among the most common consequences of thermal stress are coral bleaching together with growth, calcification and abundance decline which have lead to massive coral die-offs worldwide^4–6^.

Global warming impacts over properties and dynamics of local but also global-scale phenomena such as ENSO events^7^. Predictions state that ENSO events may increase in frequencies and severity over the next decades under conditions of climate change^8,9^. This is of great concern since these events have driven global bleaching events and mortality of coral reefs affecting the spatial refuges and viability of these ecosystems^10,11^.

The coral holobiont is an ecological unit that constitutes the coral itself and an associated microbial community (the microbiome), which includes protists, bacteria, archaea, viruses and fungi^12,13^. The microbiome, fundamental for the health and resilience of coral reefs^12,14^ plays key functions to maintain the fitness and homeostasis of the holobiont^15–17^. Microbial communities provide nutrient acquisition and disease resistance through the production of antibiotic compounds^18,19^. Additionally, the microbiome is potentially involved in different coral adaption and acclimation processes, maintaining intimate associations of high bacterial diversity with wide metabolic repertories and fast generation rates that may contribute to adaptive responses of the holobiont^20–22^. Then, being highly sensitive to environmental changes, the microbiome is capable of responding under disturbed conditions like thermal stress through microbiome restructuring^16,23–25^. The relationship between coral physiology and its microbiome may reveal why some corals are more resilient to global-scale conditions^26^. Thermal stress generates coral responses, via microbiome changes that allow the coral holobiont to increase its fitness whereas maintaining the homeostasis^16,24,27,28^. In this sense, understanding the bacterial community mechanisms that contribute to rapid coral acclimation is crucial to elucidate the holobiont responses to thermal stress under the climate change context^22^.

Gorgonian corals (Anthozoa, Octocorallia) present in shallow waters are among the most vulnerable organisms of coral ecosystems^6,29,30^. Being long-lived, sessile and slow-growing species, gorgonians are particularly sensitive to environmental changes and anthropogenic disturbances^31,32^. One example is *Pacifigorgia cairnsi*, an azooxanthellate sea fan inhabiting the remote and pristine island Malpelo located in the Colombian Tropical Eastern Pacific (TEP)^6,33,34^. This species dominates the seascape of the island forming dense aggregations on rocky reefs and walls up to 30 m depth^35,36^. Malpelo Fauna and Flora Sanctuary is a marine protected area and a World Heritage Site, considered a hot spot of biodiversity under pristine conditions^34,37^. Gorgonian populations in Malpelo cope with cold waters and high levels of suspended matter during seasonal upwelling (from December to March), as well as anomalous increases in seawater temperature during sporadic ENSO events^6,38,39^. Recently, the vulnerability of *P. cairnsi* growth rates facing anomalous increases in seawater temperature during sporadic El Niño–Southern Oscillation (ENSO) events in Malpelo has been registered^6^. This is of concern since this sea fan is considered a keystone species around the island by providing habitat and structural complexity to the benthic seascapes, and by coupling the bentho-pelagic system thanks to its strict filter feeding condition^40^.

Although numerous studies have evaluated coral’s microbiomes in general^41–44^, microbial community dynamics of corals under global-scale climate anomalies,, such as ENSO events, are not easily accessible and thus are scarce^45^. Actually, few studies of microbiome changes associated to ENSO-derived thermal anomalies affecting gorgonian corals exist^46,47^. Therefore, elucidating this kind of knowledge to gorgonian corals from remote places with absence of direct human-related stressors, as is the case of *P. cairnsi* in Malpelo Island, is highly valuable. The core microbiome of this sea fan species and its shifts associated with the necrotic patch disease (NPD) has been recently assessed^43^. However, microbiome changes driven by ENSO events of this keystone species remain elusive. Understanding micro-ecological processes, especially from the microbial component, is crucial to identify coral responses under thermal stress and hence, to evaluate the resilience capacity of these benthic organisms facing global changing conditions.

**Figure 1:**
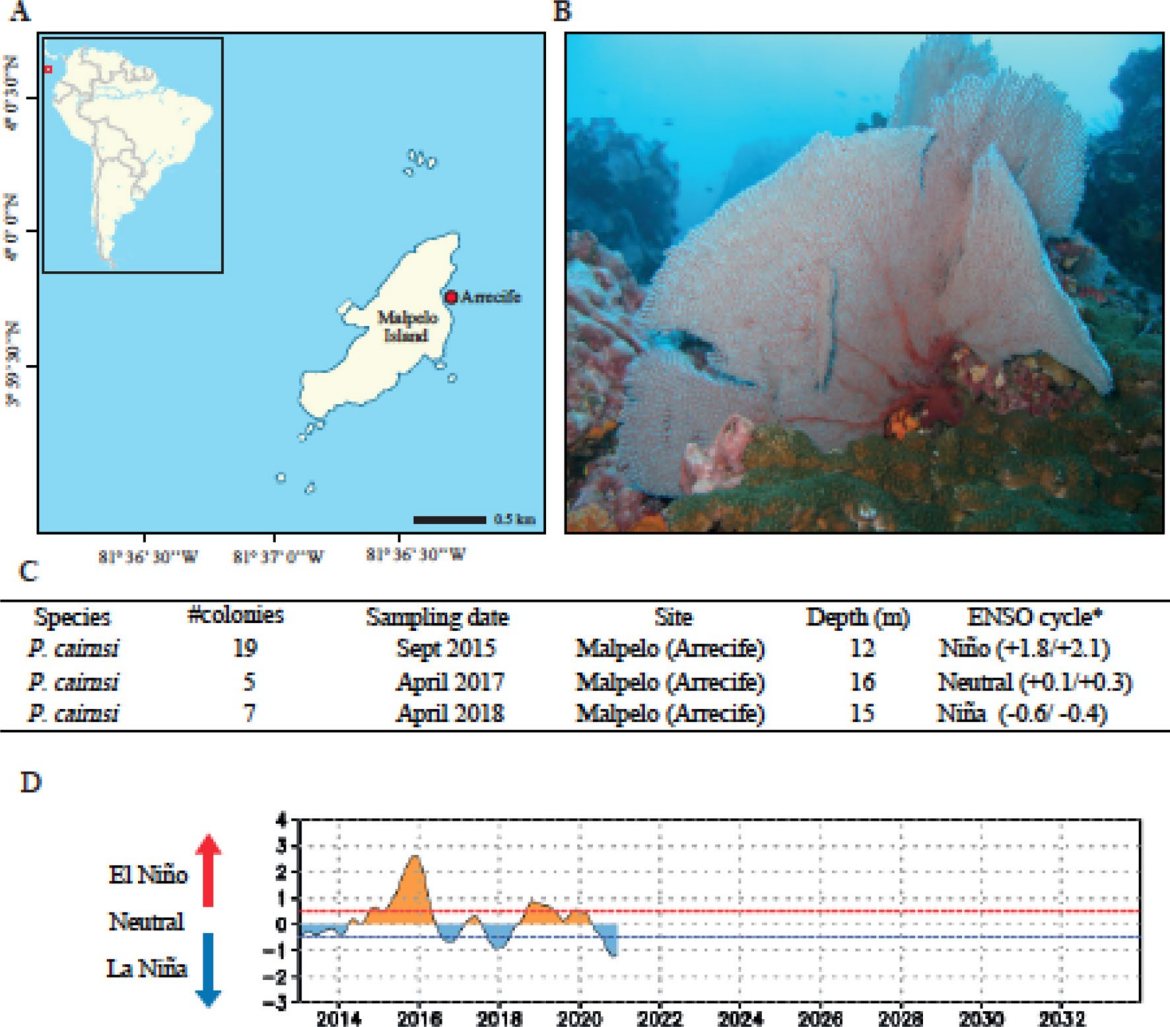
Study site and *Pacifigorgia cairnsi* sampling information. Geographical location of Malpelo Island and the sampling locality (Arrecife). Map modified from Sánchez and Ballesteros 2014, *Revista de Biología Tropical* and edited by Adobe Illustrator CS5 version 15.1.1 (**A**). *Pacifigorgia cairnsi* colony at Arrecife. Photo by Juan Armando Sánchez (**B**). Sampling information and associated ENSO phases according to the National Oceanic and Administration (NOAA *https://origin.cpc.ncep.noaa.gov) (**C**). ENSO phases dynamics between 2013 and 2021 years (**D**).

To understand the dynamics involving the holobiont and the responses to ENSO-derived thermal anomalies of corals removed from local-scale disturbances, we evaluated the composition and predicted functions of the bacterial microbiome of *P. cairnsi* sea fans through three years including different phases of ENSO cycle. This is, (1) El Niño-warming phase (year 2015), (2) La Niña-cooling phase (year 2018) and (3) the Neutral phase (year 2017) (Fig. 1). During the warm phase of ENSO (El Niño), positive anomalies of Seawater Surface Temperature (SST) are registered, while during the cooling phase of ENSO (La Niña) negative anomalies are present in the SST^48^. This study provides crucial data on how corals largely removed from direct human impacts respond to thermal stress associated with these global-scale events and may reveal why some corals are more resilient to these global change conditions. Finally, this may serve as key baseline information to understand microbiome dynamics from corals under the effects of direct-anthropogenic impacts and may provide valuable knowledge to assess coral responses according to future predictions on the climate change scenario.

## Results

16S rRNA sequences of 2015 were taken from Quintanilla et al.^43^ dataset. Specifically, we used the sequences from healthy proximal tissues in order to make it comparable to the tissues sampled in 2017 and 2018. After filtering, the final dataset consisted of 31 individual samples, 234 ASVs, a minimum of 16,760 reads and a maximum of 40,000 reads per sample and 69 recognized genera in total. Finally, 64 different ASVs accounted for the cumulative 99% of the total abundance. Rarefaction curves for all samples plateaued at 11,000 sequences, indicating a good representation of bacterial diversity (Fig. S1).

**Figure 2:**
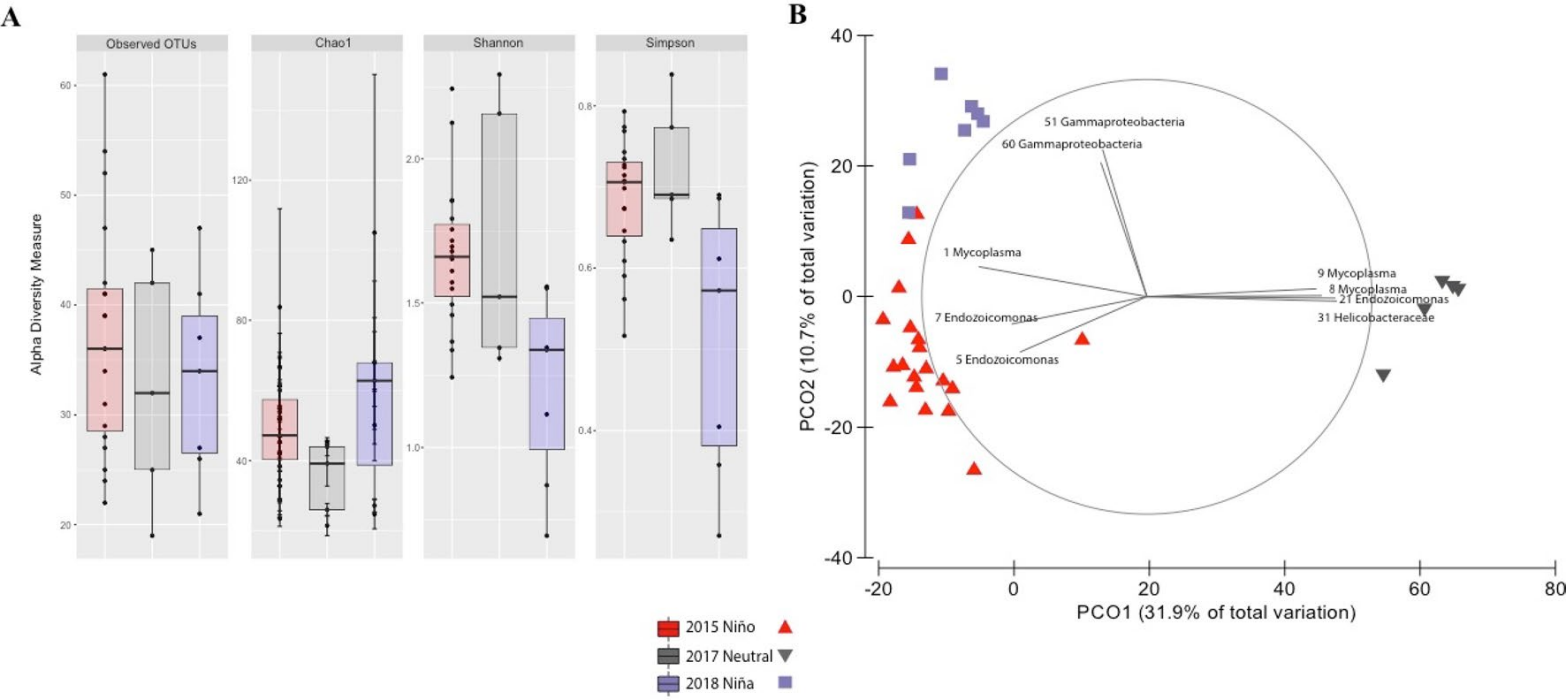
Alpha and beta-diversity metrics of *Pacifigorgia cairnsi* during sampling years. Alpha diversity metrics (number of observed ASVs, species richness Chao1, Shannon and Simpson diversity) (**A**). PCoA plot based on a Bray–Curtis dissimilarity matrix of bacterial community compositions (**B**).

Among the three years, Shannon and Simpson diversity indexes were significantly different. In the pairwise comparisons the diversity between La Niña year (2018) and the neutral year (2017) was significantly different for Simpson index and between 2018 and El Niño year (2015) for Simpson and Shannon indexes. On the other hand, Chao 1 and observed ASVs indexes did not show significant statistical differences between years (Fig. 2a; Supplementary Table 1).

Regarding beta-diversity analyses, the first two coordinates of the PCoA plot showed three clusters of samples. Samples from abnormal thermal conditions (2015 and 2018) clustered separately from samples of the neutral thermal condition (2017) (Fig. 2b). Significant differences in bacterial community compositions were obtained between each pair of compared sampling years (PERMANOVA analyses, Supplementary Table 2). The homogeneity observed in multivariate dispersions among the sampling groups (Supplementary Table 2) confirmed that the significant differences obtained with PERMANOVA were due to differences in bacterial community compositions^49^. Between years, samples of 2017 differed from those of 2015 in 92.52% and from those of 2018 with 91.41%. However, samples between 2015 and 2018 years differed 63.10% (Supplementary Table 3).

**Figure 3:**
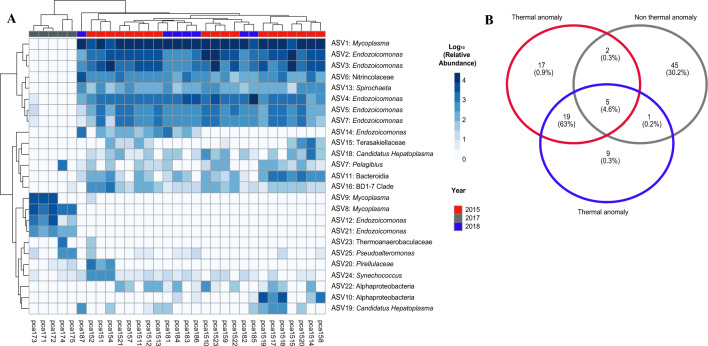
Main ASVs driving the dissimilarity and similarity between *Pacifigorgia cairnsi* colonies during sampling years. Heatmap of normalized abundances of ASVs mainly contributed to the differentiation between *Pacifigorgia cairnsi* samples according to the SIMPER analyses (**A**). Venn diagram showing the percentage of ASVs shared between *Pacifigorgia cairnsi* samples (**B**).

In general, the most abundant bacterial phylotypes were *Mycoplasma* (42.5 ± 0.3%, relative abundance of total sequences), four *Endozoicomonas* ASVs (34.1 ± 0.08%). A set of ASVs mainly conformed by *Mycoplasma* and *Endozoicomonas* bacteria members drove the differentiation between 2015 and 2018 on one hand, and 2017 on the other hand. Some of these ASVs were absent in 2017 while were highly abundant in 2015 and 2018, and the other way around (Fig. 3a, Supplementary Table S3). Specifically, in 2017 *Mycoplasma* (ASV8, ASV9), *Endozoicomonas* (ASV12 and ASV21), relative abundances were highly abundant, while in 2015 and 2018 were not present (Fig. 3a). On the other hand, in 2015 and 2018 the coral microbiome showed high relative abundances of *Mycoplasma* (ASV1), *Endozoicomonas* (ASV2, ASV3, ASV4, ASV5 and ASV7), Nitrincolaceae (ASV6) and *Spirochaeta* (ASV13). Accordingly, 2015 and 2018 shared 63% of the ASVs, while 2015 and 2017 shared 0.3%, and 2018 and 2017 shared 0.2% (Fig. 3).

**Figure 4:**
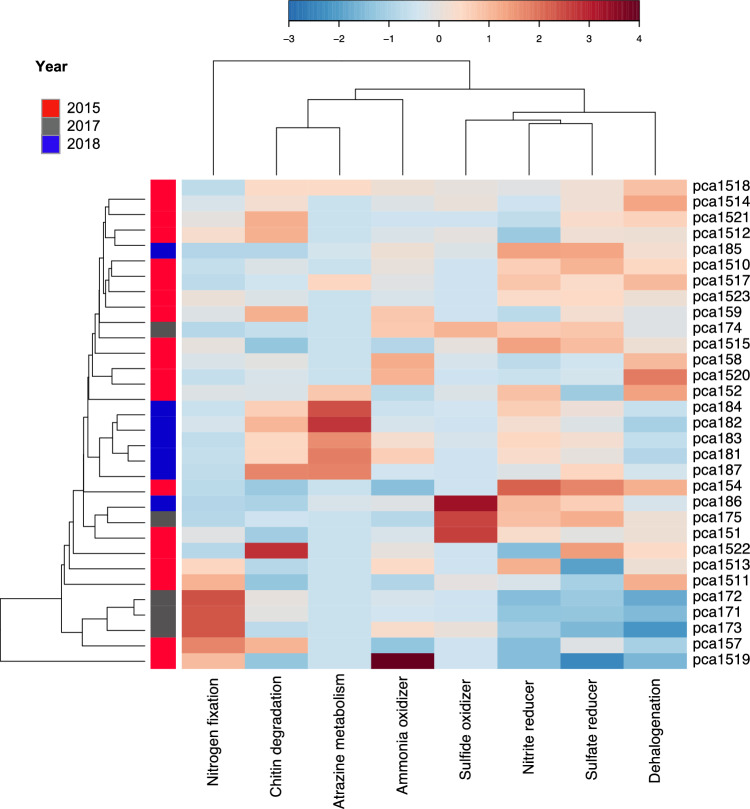
Taxonomy-based functional profiling of bacterial communities in *Pacifigorgia cairnsi* samples. Shifts in potential functional differences are represented by a relative abundance scale showing the enrichment (red colour) and depletion (blue colour) in different metabolic profiles mapped to the corresponding taxonomic information by METAGENassist. Hierarchical clustering of samples and functions was performed by a single linkage algorithm using Euclidean distance measurements.

Finally, no clustering groups were observed in predicted metabolic functions according to sampling years. A group of samples including 2015, 2017 and 2018 years were enriched in dehalogenation, ammonia oxidizer, sulfide oxidizer, sulfate reducer, nitrite reducer (Fig. 4).

## Discussion

We tested for shifts in the bacterial microbiome of gorgonian corals, without direct anthropogenic impacts, during different phases of ENSO events. Although there may exist potential limitations due to our sampling size, our results revealed similar bacterial communities between the warming (El Niño) and the cooling (La Niña) phases of the ENSO cycle, but different bacterial microbiomes between those phases, and the neutral phase. These differences were driven by a differentiation in relative abundances of a set of bacteria mainly constituted by members of *Mycoplasma* and *Endozoicomonas* taxa, with functional redundancy acting as an extended phenotype and leading acclimation of the coral under global-scale thermal anomalies, and thus potentially contributing to maintain the homeostasis at the holobiont level despite temperature regime changes.

*Pacifigorgia cairnsi* colonies showed evident changes in their microbiome structure in response to global thermal conditions derived from ENSO cycle phases. Less microbial diversity was observed in anomalous thermal years (El Niño and La Niña, 2015 and 2018, respectively) in comparison to the neutral thermal year (2017). Thermal changes affect vulnerable sessile organisms such as corals in many aspects. Diminished immune system leading to coral bleaching, disease outbreaks and dysbiosis have been linked to rising seawater temperatures^50^. Likewise, changes in coral-associated microbiomes have been observed in response to thermal stress, leading to coral die-offs^24,26,51^. The increase of bacterial diversity associated with disrupted microbiomes is a common trend found in corals affected by diseases^52,53^. In fact, microbiome shifts induced by disturbances are considered stochastic and thus dysbiotic individuals have more variability in microbial community compositions than healthy individuals in what is known as the ‘Anna Karenina principle’^54^. Increased diversity has been observed in microbiomes of heat-stressed and diseased individuals of corals such as *Orbicella faveolata, Pavona duerdeni* and *Porites lutea* and sea anemones^55–57^. However, this trend contrasts with our findings, likely indicating that *P. cairnsi* holobiont is under an acclimation process rather than a dysbiotic state.

Evident changes in microbiome compositions were observed between sampling years, revealing that thermal shifts derived from ENSO events have effects on *P. cairnsi* microbiomes. Changes of microbiome structures associated to thermal anomalies have been largely observed in corals in detriment of the holobiont’s viability^58,59^. However, these kinds of shifts affecting gorgonian corals remain largely elusive. Interestingly, we registered closer bacterial compositions between el Niño and La Niña phases (2015 and 2018, respectively) than from those in the neutral phase (2017). To our knowledge this is the first study reporting comparable in situ microbiome shifts for heat and cold stress for the same gorgonian coral species. Usually, findings are related with heat-induced microbiome shifts in detriment of coral’s viability (i.e. leading corals towards disease-prone states, coral bleaching, etc.)^59^. Some studies have shown no microbiome variations, suggesting coral’s resilience to short-term thermal stress^46,60^. Thus, our results suggest an acclimated coral holobiont not just to warm but also to cold stress by embracing similar microbiome shifts that can potentially maintain coral’s viability. The latter is evidenced by the absence of disease (NPD) symptoms and NPD-associated opportunistic microbial consortium (see Quintanilla et al.^43^).

Changes in *P. cairnsi* microbiome compositions between thermal anomalies and thermal normality mainly occurred within the same bacterial taxa. Redundancy and plasticity in core members of the microbiome may act as an extended phenotype, allowing acclimation during temperature changes^61,62^. This is, members from *Endozoicomonas*, *Mycoplasma* and *Spirochaeta* taxonomic groups were contrarily present in the warming and cooling phase of ENSO on one side, and in the neutral phase on the other side. Interestingly, *Endozoicomonas* and *Mycoplasma* bacterial members were found to be dominant core microbiome members of healthy *P. cairnsi* colonies^43^ and are known to typically constitute dominant endosymbionts in scleractinian and gorgonian core microbiomes^42,63,64^. Some *Endozoicomonas* produce antimicrobial compounds against pathogens, thus contributing to maintain the coral health state^19,65^. *Mycoplasma*, although is a dominant member of the microbiome of healthy colonies of gorgonians and cold-water scleractinians, its specific role within the coral holobiont remains unclear^43,47^. Therefore, the fact that we found an enrichment of specific *Endozoicomonas* and *Mycoplasma* members in corals under thermal anomalies may be indicative of possible protection of the holobiont against environmental-thermal stress. Moreover, Nitrincolaceae is a family found enriched in disease corals and hypoxic coral samples^66,67^, environments under stress conditions. These findings are consistent with the high levels of Nitrincolaceae members found in the abnormal thermal years, the cool and warm phases of ENSO. Finally, *Spirochaeta* is a chemioheterotrophic bacteria found in different environments^68^. Spirochaetes members are reported in healthy corals such as *Muricea californica*^42^, *Paragorgia arborea*^69^, *Corallium rubrum*^70^, *Thouarella superba*^71^ but also in diseased corals^55,72,73^. Members of Spirochaetes were suggested as potential nitrogen fixers^74^ and carbon fixers^75^. We found *Spirochaeta* in all samples of thermal anomalies (2015, 2018) in higher abundances than in neutral thermal conditions (2017). Due to its important role in host metabolism^76^, it is probable that this bacteria is key in restructuring the *P. cairnsi* microbiome in order to prevent a dysbiosis state during thermal anomalies.

**Figure 5:**
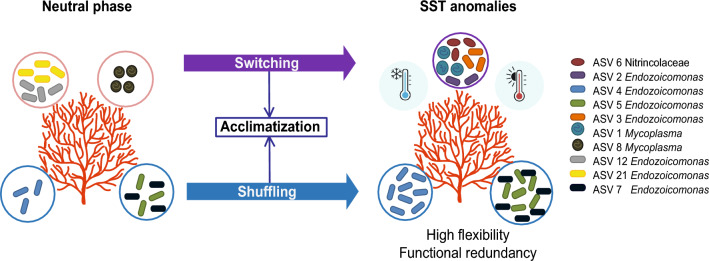
Proposed bacterial community dynamics potentially supporting holobiont acclimation of *Pacifigorgia cairnsi* via shuffling (blue) and switching (purple). Coral holobiont under neutral phase maintains specific microorganisms. The microbial shifts help the coral to acquire more flexibility under SST anomalies (El Niño and La Niña phases) and acclimate via functional redundancy. Labels of relevant bacterial members driving microbiome shifts and acclimation and their corresponding ASV numbers are shown in right.

*Pacifigorgia cairnsi* harbouring different bacterial members from the same taxonomic group under thermal anomalies may indicate that the holobiont is choosing a best-suited microbiome to face the environmental disturbances. Three concepts are related with the latter. The first one, the ‘Coral Probiotic hypothesis’, which states that corals can improve coral health and resilience by shifting the abundance and diversity of their microbial partners and thus selecting the most advantageous coral holobiont in response to changing environmental conditions^77^. The second concept, the ‘symbiont switching’, which states that coral acquires novel bacteria from the environmental pool under altered environmental conditions, triggering coral acclimation^78^. For instance, symbiont switching to thermally resistant *Symbiodinium* clades has been reported in zooxanthellate corals^79–82^. Additionally, some bacteria have been registered to facilitate resilience in corals during warm temperature anomalies^77,83^. The third concept related with our results is ‘symbiont shuffling’, which states that microbial frequency shifts are observed during environmental stress to acclimate the coral holobiont^78^. See Fig. 5. Specifically, *Endozoicomonas* (ASV4, ASV5 and ASV7) were enriched in the SST anomaly years compared with the neutral phase (Fig. 5). In fact, proliferation of *Endozoicomonas* after warm periods has been related to the ability to mitigate holobiont dysbiosis to thermal stress and to the acclimation and resilience capacity of coral microbiomes^84^. Therefore, these azooxanthellate sea fans may be extending their phenotypes by selecting the most advantageous holobiont under different ENSO phases (warming and cooling) by shifting the structure and diversity of associated bacterial communities of their core microbiome for others of the same taxon, thus allowing the acclimation and adaptation of the holobiont under thermal stress.

Results in the microbiome composition are consistent with the trend observed in the predicted functional profiling of the microbiome. Although the microbiome composition changed, no differences were observed in the predicted functionality between the three years of the study. Vital functions for the viability of the coral holobiont^85^, such as those related with nitrogen and sulfur cycles seem to be equally expressed in *P. cairnsi* microbiomes in neutral year and thermal anomaly years. This suggests that *P. cairnsi* exhibits metabolic redundancy in their microbiome despite its composition changes under thermal stress, meaning that different bacterial species fulfil similar functions within the coral holobiont. By shifting some core microbiome members (potentially for more thermally resistant ones), the sea fan secures to maintain the core microbiome functions and thus achieves holobiont plasticity and stability for rapid acclimation under thermal anomalies^28,86–89^. Additionally, the fact that these are azooxanthellate corals (i.e., no dependency on autotrophy) could also support their acclimation potential and resilient capacity to environmental changes^90,91^. The patterns found here are particularly interesting since numerous reef-building coral species worldwide have shown vulnerability and decay patterns when facing thermal stress^92–94^. Therefore, our findings highlight that resilience and adaptability capacity of gorgonian corals, possibly enhanced by the pristine conditions of the environment.

Overall, our results suggest coral holobiont acclimation not just to thermal but also to cold stress by embracing similar microbiome shifts. These findings may be indicating that this coral holobiont has a well-established relationship with their microbial partners, all together potentially representing a solid acclimated and resilient mechanism under thermal and cold stress.

By restructuring microbial communities and by maintaining functional redundancy, the gorgonian holobiont shows adaptation to thermal stress derived from global-scale disturbances such as ENSO events. This study reveals that the coral microbiome has the ability to adapt rapidly, acting as an extended plastic phenotype, during thermal stress under pristine conditions in order to maintain *fitness* at the holobiont level.

In further studies we encourage to increase sample size and to incorporate omic approaches (e.g. metagenomics and metatranscriptomics) in order deepen and confirm microbiome mechanisms of *P. cairnsi* acclimation to ENSO-derived thermal anomalies. We also suggest studying abiotic changes related to ENSO events that may also modulate the microbiome shifts in order to obtain an overview of different processes that may affect the response of the coral holobiont under thermal stress conditions. Finally, we suggest assessing coral microbiome dynamics under future predictions of ENSO events in order to understand ongoing responses and the adaptability threshold of coral holobionts under climate change scenarios.

## Materials and methods

### Study site and sample collection

*Pacifigorgia cairnsi* sampling was conducted in Malpelo (Arrecife reef), an oceanic remote island of difficult access about 500 km off the Colombian coast in the Tropical Eastern Pacific (3° 58′ 30″ N, 81° 34′ 48″ W) (Fig. 1). Malpelo Fauna and Flora Sanctuary is a marine protected area and a World Heritage Site, considered a hot spot of biodiversity under pristine conditions^34,37^. Samples were collected in September 2015 (n = 19), April 2017 (n = 5) and April 2018 (n = 7) (Fig. 1). Due to the remote condition and difficult access to Malpelo Island, having a large number of samples from different years, is particularly valuable. Sampling description is detailed in Quintanilla et al.^43^. After collection each sample was gently rinsed with 100 ml filtered fresh water to remove transient microorganisms loosely associated with the coral tissue. All samples were taken from colonies visibly healthy (i.e. absence of NPD symptoms, see Quintanilla et al.^43^). Samples were stored in RNAlater (Thermo Fisher Scientific, Waltham, USA) for 2015 samples and in DMSO for 2017 and 2018. All samples were preserved at − 80 °C until subsequent DNA extraction. Collections were made possible with research permit No. 105 (2013), issued by the Autoridad Nacional de Licencias Ambientales-ANLA, Ministerio de Ambiente y Desarrollo Sostenible, Colombia, Contrato de Acceso a Recursos Genéticos para Investigación Científica Sin Interés Comercial No. 106, 20 (2014) RGE0114 [1] and through resolution 1177 of October 9th, 2014-IBD 0359 from Universidad de los Andes.

Regarding global thermal conditions and according to the National Oceanic and Administration (NOAA) (http://www.cpc.ncep.noaa.gov), September 2015 corresponded to the El Niño-warming phase warming phase (+ 1.8/ + 2.1 °C), April 2017 corresponded to the Neutral phase (+ 0.1/ + 0.3 °C) and April 2018 to La Niña-cooling phase (− 0.6/− 0.4 °C). See Fig. 1. Sea surface temperatures were provided by Fundación Malpelo.

### DNA extraction and 16S rRNA gene sequencing

Sequences of 2015 were taken from Quintanilla et al.^43^ (peripheral tissues from healthy colonies-HP). Sequences of 2017 and 2018 were obtained following those author’s descriptions. In brief, the variable V4 region of the 16S rRNA gene was sequenced using the 515F/806R PCR primers and Illumina flowcell adapter sequences according to the earth microbiome protocol^95^ (http://www.earthmicrobiome.org/emp-standard-protocols/16s/). The Takara Taq DNA polymerase premix was used for PCR amplifications as referred in Quintanilla et al.^43^. Barcoded amplicons were pooled and sequenced on the Illumina MiSeq platform (Illumina, San Diego, USA), implementing 2 × 250 bp paired end read libraries.

### Microbiome data analyses

Amplicon sequence data were demultiplexed, assembled and analysed using QIIME2 version 2023.5)^96^ to identify Amplicon Sequence Variants (ASVs). Denoising, chimera filtering, and trimming was performed in Deblur^97^. Taxonomy was assigned using the trained classifier SILVA 138 at 99% of similarity (silva-138-nb-classifer)^98^. Singleton ASVs were removed to minimize false ASVs, as well as ASVs identified as eukaryotes, mitochondria or chloroplasts. Subsequently, the table was filtered to remove uninformative and low quality features to keep ASVs with at least 3 counts in at least 3 samples (~ 10% of the total samples).

Alpha diversity metrics analysis (total number of observed ASVs, species richness Chao1, Shannon and Simpson diversity index) and rarefaction curves were generated through phyloseq package^99^ in R version 4.3.1 in order to obtain a comprehensive description of the within-sample bacterial community. Differences in alpha diversity metrics between sampling years were tested with Kruskal–Wallis and ANOVA tests.

To assess differences in bacterial community compositions between samples from different sampling years (beta diversity) we considered taxa accounting for the cumulative 99% of total abundance. We conducted multivariate analyses using PRIMER 6 & PERMANOVA+ software^100^. In order to visualize differences in bacterial community compositions between sampling years, principal coordinate analysis (PCoA) was performed, applying a square-root transformation to relative abundances and calculating Bray–Curtis dissimilarity matrices. Permutational Analyses of Variance (PERMANOVA) were conducted to test differences in bacterial community compositions between sampling years (9999 permutations). Additionally, Permutational Analysis of Multivariate Dispersions (PERMDISP) were used to test for homogeneity of multivariate dispersions (9999 permutations) between sampling groups. Similarity percentage (SIMPER) analyses were used to identify the taxa contributing to the greatest extent to the observed patterns, taking into account a cutoff greater than 1% of contribution. Accordingly, a heat map was elaborated using the package phyloseq with the function *plot_taxa_heatmap* using log10 as normalizing parameter. Venn diagrams showing shared and unique ASVs per sampling year (percentage of the relative abundances) were performed using the package ampvis2 with the function *amp_venn* with an abundance cutoff of 0.1 and frequency cutoff of 10.

Putative functional differences associated with shifts in bacterial community compositions among sampling years were assessed by using METAGENassist^101^. ASVs filtering and normalization parameters were used as described by^102^. Euclidean distance measure (single linkage algorithm) was used to visualize functional profiles in heatmap mapped to the microbial communities.

### Accession numbers

The sequences were deposited in the NCBI Sequence Read Archive (SRA) under BioProject number PRJNA403829 for 2015, BioProject number PRJNA722752 for 2017 samples (SAMN18791299-SAMN18791303) and Bioproject number PRJNA737787 for 2018 samples (SAMN19712929-SAMN19712935).

### Supplementary Information

Supplementary Information.

## Data Availability

All data are available in the main text or the supplementary materials. The sequences were deposited in the NCBI Sequence Read Archive (SRA) under BioProject number PRJNA403829 for 2015, BioProject number PRJNA722752 for 2017 samples (SAMN18791299-SAMN18791303) and Bioproject number PRJNA737787 for 2018 samples (SAMN19712929-SAMN19712935).
