# Supplementary material for: Microbial shifts associated to ENSO-derived thermal anomalies reveal coral acclimation at holobiont level
Source: Sci Rep. 2023 Dec 12;13:22049. doi: 10.1038/s41598-023-49049-6 (PMC10716379; doi:10.1038/s41598-023-49049-6)
Supplement: Supplementary file 1 — Supplementary Information. [file 41598_2023_49049_MOESM1_ESM.pdf]

# Supplementary Materials for

## **Microbial shifts associated to ENSO-derived thermal anomalies reveal coral acclimation at holobiont level**

Sandra Montaño-Salazar, Elena Quintanilla,* and Sánchez J. A

*Corresponding author. Email: elena.quintanilla8@gmail.com

**Fig. S1.**

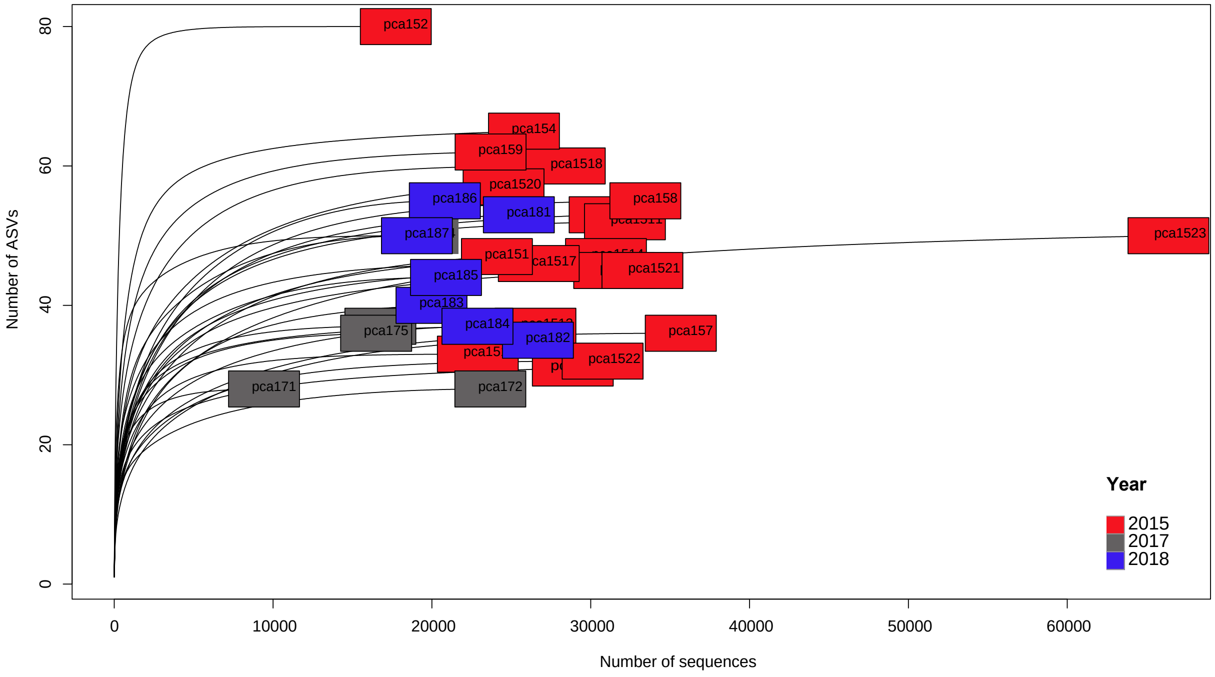


Rarefaction curves for the ASVs of *Pacifigorgia cairnsi* samples from the three sampling years.

**Table S1.**

**a)**

| Kruskal test | Chi-square | diff | p-value |
|---|---|---|---|
| Simpson | 7.873 | 2 | 0.01952 |
| Shannon | 7.891 | 2 | 0.01934 |

| Pairwise Wilcoxon rank | Simpson | Shannon |
|---|---|---|
| 2017-2015 | 0.534 | 0.8906 |
| 2018-2015 | 0.025 | 0.0066 |
| 2018-2017 | 0.045 | 0.303 |

**b)**

| Tukey test Observed ASVs | diff | lwr | upr | p adj |
|---|---|---|---|---|
| 2017-2015 | -4.663 | -17.694 | 8.368 | 0.653 |
| 2018-2015 | -3.977 | -15.44 | 7.486 | 0.67 |
| 2018-2017 | 0.685 | -14.495 | 15.867 | 0.993 |

| Tukey test Chao1 | diff | lwr | upr | p adj |
|---|---|---|---|---|
| 2017-2015 | -13.422 | -35.525 | 8.68 | 0.305 |
| 2018-2015 | 9.414 | -10.029 | 28.857 | 0.464 |
| 2018-2017 | 22.836 | -2.9123 | 48.586 | 0.089 |

Comparison test for Shannon and Simpson diversity metrics (a) and for Chao 1 and observed ASVs (b) of *Pacifigorgia cairnsi* microbiomes in the three sampling years.

**Table S2**

| | | | PERMANOVA | | | PERMANOVA Pair-wise tests | | | PERMDISP | | |
|---|---|---|---|---|---|---|---|---|---|---|---|
| Source | df | SS | MS | Pseudo-F | P (perm) | Groups | t | P (perm) | Groups | t | P (perm) |
| Sampled years | 2 | 29512 | 14756 | 9.154 | 0.0001* | 2015, 2017 | 3.64 | 0.0001* | 2015, 2017 | 1.04 | 0.5125 |
| Res | 28 | 45134 | 1611.9 | | | 2015, 2018 | 2.03 | 0.0001* | 2015, 2018 | 1.91 | 0.1334 |
| Total | 30 | 74646 | | | | 2017, 2018 | 3.43 | 0.0015* | 2017, 2018 | 0.501 | 0.687 |

Permanova and Permdisp analyses and pair-wise comparisons between sampling years.

**Table S3.**

Groups 2018 & 2017
Average dissimilarity = 92.52

| | Group 2018 | Group 2017 | | | | |
|---|---|---|---|---|---|---|
| ASVs | Av.Abund | Av.Abund | Av.Diss | Diss/SD | Contrib% | Cum.% |
| ASV21 | 0 | 4.3 | 4.04 | 2.34 | 4.36 | 4.36 |
| ASV8 | 0 | 4.21 | 3.98 | 2.08 | 4.3 | 8.66 |
| ASV31 | 0 | 4.24 | 3.85 | 2.88 | 4.16 | 12.83 |
| ASV9 | 0.02 | 3.45 | 3.59 | 1.18 | 3.88 | 16.71 |
| ASV12 | 0 | 3.46 | 3.55 | 1.1 | 3.84 | 20.55 |
| ASV38 | 0 | 3.5 | 3.05 | 1.27 | 3.3 | 23.85 |
| ASV28 | 1 | 2.54 | 2.35 | 1.14 | 2.54 | 26.39 |
| ASV57 | 0.12 | 3.01 | 2.32 | 0.92 | 2.51 | 28.9 |
| ASV44 | 0.2 | 2.91 | 2.28 | 0.97 | 2.46 | 31.36 |
| ASV25 | 0.29 | 2.78 | 2.12 | 0.9 | 2.29 | 33.65 |
| ASV52 | 0.7 | 2.83 | 2.05 | 1.36 | 2.22 | 35.87 |
| ASV61 | 0.13 | 2.56 | 1.94 | 0.79 | 2.1 | 37.97 |
| ASV2 | 2.09 | 0 | 1.94 | 2.15 | 2.09 | 40.06 |
| ASV3 | 2.04 | 0 | 1.87 | 2.12 | 2.02 | 42.08 |
| ASV13 | 1.93 | 0 | 1.79 | 2.25 | 1.94 | 44.02 |
| ASV1 | 1.91 | 0 | 1.78 | 2.74 | 1.93 | 45.95 |
| ASV5 | 1.99 | 0.07 | 1.78 | 2.23 | 1.93 | 47.88 |
| ASV7 | 1.92 | 0.03 | 1.77 | 2.06 | 1.91 | 49.79 |
| ASV17 | 1.07 | 1.61 | 1.71 | 1.07 | 1.84 | 51.63 |

| | | | | | | |
|---|---|---|---|---|---|---|
| ASV41 | 1.09 | 2.66 | 1.63 | 1.6 | 1.76 | 53.39 |
| ASV11 | 1.83 | 0 | 1.6 | 1.38 | 1.73 | 55.12 |
| ASV56 | 0.39 | 2.07 | 1.57 | 0.76 | 1.69 | 56.82 |
| ASV16 | 1.83 | 0.06 | 1.53 | 1.5 | 1.65 | 58.47 |
| ASV6 | 1.67 | 0 | 1.53 | 2.35 | 1.65 | 60.12 |
| ASV33 | 1.66 | 0 | 1.51 | 1.28 | 1.63 | 61.74 |
| ASV53 | 0.5 | 1.21 | 1.44 | 0.87 | 1.56 | 63.3 |
| ASV26 | 0.86 | 1.49 | 1.44 | 0.82 | 1.56 | 64.86 |
| ASV22 | 1.5 | 0 | 1.44 | 0.89 | 1.55 | 66.41 |
| ASV4 | 1.54 | 0.02 | 1.41 | 2.42 | 1.52 | 67.93 |
| ASV27 | 1.47 | 0 | 1.36 | 0.84 | 1.47 | 69.4 |
| ASV18 | 1.51 | 0 | 1.36 | 0.88 | 1.47 | 70.87 |
| ASV50 | 0.17 | 1.95 | 1.35 | 0.56 | 1.46 | 72.33 |
| ASV43 | 0.18 | 1.93 | 1.34 | 0.55 | 1.45 | 73.78 |
| ASV23 | 0.14 | 1.94 | 1.3 | 0.53 | 1.4 | 75.19 |
| ASV37 | 1.49 | 0 | 1.24 | 0.91 | 1.34 | 76.53 |
| ASV32 | 0.93 | 1.17 | 1.24 | 1.42 | 1.34 | 77.87 |
| ASV15 | 1.15 | 0 | 1.06 | 0.57 | 1.14 | 79.01 |
| ASV29 | 1.19 | 0 | 0.97 | 0.7 | 1.05 | 80.06 |
| ASV55 | 1.19 | 0 | 0.96 | 0.68 | 1.04 | 81.1 |
| ASV46 | 1.24 | 0 | 0.96 | 0.79 | 1.03 | 82.13 |
| ASV35 | 0.84 | 0.4 | 0.95 | 0.44 | 1.02 | 83.15 |
| ASV10 | 1.03 | 0 | 0.92 | 0.47 | 1 | 84.15 |
| ASV30 | 1.04 | 0 | 0.92 | 0.59 | 0.99 | 85.14 |
| ASV49 | 1.04 | 0 | 0.92 | 0.5 | 0.99 | 86.13 |
| ASV47 | 1.04 | 0 | 0.9 | 0.5 | 0.97 | 87.11 |
| ASV34 | 0.95 | 0.42 | 0.87 | 0.71 | 0.94 | 88.05 |
| ASV24 | 1.34 | 0.61 | 0.83 | 0.65 | 0.9 | 88.95 |
| ASV48 | 0.77 | 0.47 | 0.82 | 0.67 | 0.89 | 89.84 |
| ASV19 | 0.94 | 0 | 0.82 | 0.61 | 0.88 | 90.72 |

Groups 2015 & 2018

Average dissimilarity = 63.10

| ASVs | Group 2015 Av.Abund | Group 2018 Av.Abund | Av.Diss | Diss/SD | Contrib% | Cum.% |
|---|---|---|---|---|---|---|
| ASV60 | 0.46 | 3.1 | 2.64 | 1.93 | 4.19 | 4.19 |
| ASV64 | 0.57 | 2.66 | 2.55 | 2.29 | 4.04 | 8.23 |
| ASV51 | 0.74 | 2.89 | 2.35 | 1.67 | 3.73 | 11.96 |
| ASV14 | 0.73 | 2.2 | 2.09 | 1.08 | 3.31 | 15.27 |
| ASV42 | 0.56 | 1.82 | 1.86 | 0.84 | 2.95 | 18.22 |
| ASV63 | 0.82 | 1.59 | 1.79 | 1.05 | 2.83 | 21.05 |
| ASV19 | 0.94 | 1.25 | 1.61 | 0.84 | 2.55 | 23.61 |
| ASV22 | 1.5 | 0.56 | 1.58 | 1.02 | 2.5 | 26.11 |
| ASV36 | 0.53 | 1.43 | 1.58 | 0.62 | 2.5 | 28.61 |
| ASV58 | 0.29 | 1.55 | 1.54 | 0.58 | 2.44 | 31.05 |
| ASV27 | 1.47 | 0.46 | 1.51 | 0.92 | 2.4 | 33.45 |
| ASV2 | 2.09 | 0.69 | 1.45 | 1.61 | 2.3 | 35.74 |
| ASV33 | 1.66 | 1.1 | 1.42 | 1.36 | 2.25 | 37.99 |
| ASV11 | 1.83 | 0.47 | 1.4 | 1.25 | 2.22 | 40.21 |
| ASV37 | 1.49 | 0.12 | 1.33 | 0.95 | 2.11 | 42.32 |
| ASV30 | 1.04 | 0.78 | 1.32 | 0.85 | 2.09 | 44.41 |
| ASV18 | 1.51 | 0.81 | 1.3 | 0.93 | 2.06 | 46.47 |
| ASV16 | 1.83 | 0.49 | 1.27 | 1.2 | 2.02 | 48.49 |
| ASV3 | 2.04 | 0.87 | 1.24 | 1.4 | 1.96 | 50.45 |
| ASV4 | 1.54 | 2.37 | 1.2 | 1.33 | 1.9 | 52.35 |
| ASV55 | 1.19 | 0.49 | 1.2 | 0.85 | 1.89 | 54.25 |
| ASV15 | 1.15 | 0.03 | 1.16 | 0.58 | 1.83 | 56.08 |
| ASV29 | 1.19 | 0.31 | 1.14 | 0.8 | 1.81 | 57.89 |
| ASV41 | 1.09 | 0.82 | 1.12 | 0.97 | 1.78 | 59.66 |
| ASV6 | 1.67 | 1.94 | 1.11 | 0.94 | 1.75 | 61.42 |
| ASV10 | 1.03 | 0.11 | 1.05 | 0.51 | 1.66 | 63.08 |
| ASV47 | 1.04 | 0.13 | 1.03 | 0.55 | 1.63 | 64.71 |
| ASV24 | 1.34 | 0.99 | 1.03 | 0.85 | 1.63 | 66.34 |

| | | | | | | |
|---|---|---|---|---|---|---|
| ASV32 | 0.93 | 0.57 | 1.02 | 0.87 | 1.61 | 67.95 |
| ASV52 | 0.7 | 0.85 | 1.01 | 1.08 | 1.6 | 69.55 |
| ASV49 | 1.04 | 0 | 1.01 | 0.51 | 1.59 | 71.14 |
| ASV46 | 1.24 | 0.46 | 1.01 | 0.89 | 1.59 | 72.73 |
| ASV62 | 0.98 | 0.27 | 0.99 | 0.58 | 1.58 | 74.31 |
| ASV35 | 0.84 | 0.16 | 0.98 | 0.42 | 1.55 | 75.86 |
| ASV17 | 1.07 | 0.25 | 0.96 | 1.3 | 1.52 | 77.38 |
| ASV28 | 1 | 0.65 | 0.96 | 1.22 | 1.52 | 78.9 |
| ASV54 | 0.67 | 0.52 | 0.95 | 0.68 | 1.5 | 80.4 |
| ASV26 | 0.86 | 0.52 | 0.93 | 1 | 1.47 | 81.88 |
| ASV5 | 1.99 | 1.4 | 0.86 | 1.43 | 1.36 | 83.23 |
| ASV7 | 1.92 | 1.53 | 0.84 | 1.39 | 1.33 | 84.57 |
| ASV61 | 0.13 | 0.82 | 0.84 | 0.85 | 1.33 | 85.9 |
| ASV13 | 1.93 | 1.59 | 0.78 | 1.22 | 1.24 | 87.14 |
| ASV40 | 0.95 | 0 | 0.78 | 0.49 | 1.23 | 88.37 |
| ASV34 | 0.95 | 0 | 0.77 | 0.51 | 1.21 | 89.58 |
| ASV45 | 0.73 | 0.26 | 0.75 | 0.51 | 1.2 | 90.78 |

Groups 2017 & 2018
Average dissimilarity = 91.41

| ASVs | Group 2017 Av.Abund | Group 2018 Av.Abund | Av.Diss | Diss/SD | Contrib% | Cum.% |
|---|---|---|---|---|---|---|
| ASV21 | 4.3 | 0 | 4.51 | 2.41 | 4.94 | 4.94 |
| ASV8 | 4.21 | 0 | 4.45 | 2.13 | 4.87 | 9.8 |
| ASV9 | 3.45 | 0 | 4.05 | 1.19 | 4.43 | 14.23 |
| ASV12 | 3.46 | 0 | 4 | 1.11 | 4.37 | 18.6 |
| ASV31 | 4.24 | 0.44 | 3.82 | 2.65 | 4.18 | 22.78 |
| ASV38 | 3.5 | 0 | 3.39 | 1.29 | 3.71 | 26.48 |
| ASV60 | 0 | 3.1 | 3.08 | 2.28 | 3.37 | 29.85 |
| ASV51 | 0 | 2.89 | 2.94 | 1.92 | 3.22 | 33.07 |
| ASV64 | 0 | 2.66 | 2.76 | 2.73 | 3.01 | 36.09 |

| | | | | | | |
|---|---|---|---|---|---|---|
| ASV28 | 2.54 | 0.65 | 2.75 | 1.12 | 3.01 | 39.1 |
| ASV57 | 3.01 | 0 | 2.55 | 0.91 | 2.79 | 41.89 |
| ASV44 | 2.91 | 0 | 2.49 | 0.96 | 2.73 | 44.62 |
| ASV61 | 2.56 | 0.82 | 2.39 | 1.03 | 2.61 | 47.23 |
| ASV4 | 0.02 | 2.37 | 2.35 | 1.84 | 2.57 | 49.8 |
| ASV52 | 2.83 | 0.85 | 2.33 | 1.45 | 2.55 | 52.35 |
| ASV25 | 2.78 | 0.36 | 2.31 | 0.92 | 2.53 | 54.88 |
| ASV14 | 0 | 2.2 | 2.23 | 0.95 | 2.44 | 57.32 |
| ASV1 | 0 | 1.93 | 2.01 | 2.74 | 2.19 | 59.51 |
| ASV6 | 0 | 1.94 | 1.99 | 1.26 | 2.17 | 61.69 |
| ASV41 | 2.66 | 0.82 | 1.87 | 1.86 | 2.05 | 63.74 |
| ASV42 | 0 | 1.82 | 1.69 | 0.68 | 1.85 | 65.59 |
| ASV63 | 0 | 1.59 | 1.61 | 0.92 | 1.76 | 67.35 |
| ASV13 | 0 | 1.59 | 1.6 | 2.43 | 1.75 | 69.1 |
| ASV7 | 0.03 | 1.53 | 1.54 | 2.16 | 1.69 | 70.79 |
| ASV56 | 2.07 | 0 | 1.51 | 0.67 | 1.65 | 72.44 |
| ASV50 | 1.95 | 0.18 | 1.47 | 0.56 | 1.61 | 74.05 |
| ASV26 | 1.49 | 0.52 | 1.42 | 0.77 | 1.56 | 75.61 |
| ASV58 | 0 | 1.55 | 1.38 | 0.49 | 1.51 | 77.12 |
| ASV5 | 0.07 | 1.4 | 1.36 | 2.35 | 1.48 | 78.61 |
| ASV36 | 0 | 1.43 | 1.34 | 0.48 | 1.47 | 80.07 |
| ASV23 | 1.94 | 0 | 1.33 | 0.49 | 1.46 | 81.53 |
| ASV43 | 1.93 | 0 | 1.33 | 0.49 | 1.45 | 82.98 |
| ASV17 | 1.61 | 0.25 | 1.29 | 0.6 | 1.41 | 84.39 |
| ASV19 | 0 | 1.25 | 1.25 | 0.6 | 1.37 | 85.76 |
| ASV53 | 1.21 | 0.13 | 1.18 | 1.2 | 1.29 | 87.05 |
| ASV33 | 0 | 1.1 | 1.17 | 0.91 | 1.28 | 88.33 |
| ASV3 | 0 | 0.87 | 0.87 | 2.67 | 0.95 | 89.28 |
| ASV18 | 0 | 0.81 | 0.83 | 1.12 | 0.91 | 90.19 |

| Taxonomy | |
|---|---|
| ASV1 | Mycoplasma |
| ASV2 | Endozoicomonas |
| ASV3 | Endozoicomonas |
| ASV4 | Endozoicomonas |
| ASV5 | Endozoicomonas |
| ASV6 | Nitrincolaceae |
| ASV7 | Endozoicomonas |
| ASV8 | Mycoplasma |
| ASV9 | Mycoplasma |
| ASV10 | Alphaproteobacteria |
| ASV11 | Bacteroidia |
| ASV12 | Endozoicomonas |
| ASV13 | Spirochaeta_2 |
| ASV14 | Endozoicomonas |
| ASV15 | Terasakiellaceae |
| ASV16 | Spongiibacteraceae |
| ASV17 | Pelagibius |
| ASV18 | Candidatus_Hepatoplasma |
| ASV19 | Candidatus_Hepatoplasma |
| ASV20 | Pirellulaceae |
| ASV21 | Endozoicomonas |
| ASV22 | Alphaproteobacteria |
| ASV23 | Thermoanaerobaculaceae |
| ASV24 | Synechococcus_CC9902 |
| ASV25 | Pseudoalteromonas |
| ASV26 | Rhodobacteraceae |
| ASV27 | Pelagibius |
| ASV28 | BD72BR169 |
| ASV29 | Alphaproteobacteria |

| | |
|---|---|
| ASV30 | Kordia |
| ASV31 | Helicobacteraceae |
| ASV32 | Cyanobium_PCC-6307 |
| ASV33 | Spirochaetaceae |
| ASV34 | Staphylococcus |
| ASV35 | Vibrio |
| ASV36 | Rhodobacteraceae |
| ASV37 | Pelagibius |
| ASV38 | Endozoicomonas |
| ASV39 | Alphaproteobacteria |
| ASV40 | Thermoanaerobaculaceae |
| ASV41 | Vibrio |
| ASV42 | Endozoicomonas |
| ASV43 | Gammaproteobacteria |
| ASV44 | Algicola |
| ASV45 | Stappiaceae |
| ASV46 | Synechococcus_CC9902 |
| ASV47 | Aliikangiella |
| ASV48 | Streptococcus |
| ASV49 | Ketobacter |
| ASV50 | Labrenzia |
| ASV51 | Labrenzia |
| ASV52 | Vibrio |
| ASV53 | Escherichia-Shigella |
| ASV54 | Methylobacterium-Methylorubrum |
| ASV55 | Anderseniella |
| ASV56 | Aestuariibacter |
| ASV57 | Pseudoalteromonas |
| ASV58 | Pseudoalteromonas |
| ASV59 | Rhizobiaceae |
| ASV60 | Gammaproteobacteria |
| ASV61 | Alteromonas |

| | |
|---|---|
| ASV62 | Alteromonas |
| ASV63 | Terasakiellaceae |
| ASV64 | Pseudomonas |

Similarity Percentage analyses (SIMPER) and ASVs taxonomical identification (highest level of taxonomic resolution is shown for each ASVs). Main ASVs driving similarities in bacterial community compositions of *Pacifigorgia cairnsi* within and between sampling years. Results explained >90% of the similarity observed**.**
